# Investigations on the Interaction Behavior between Direct Reduced Iron and Various Melts

**DOI:** 10.3390/ma15165691

**Published:** 2022-08-18

**Authors:** Andreas Pfeiffer, Gerald Wimmer, Johannes Schenk

**Affiliations:** 1 Department of Metallurgy, Chair of Ferrous Metallurgy, Montanuniversität Leoben, Franz-Josef-Straße 18, 8700 Leoben, Austria; 2 Primetals Technologies Austria, 4031 Linz, Austria

**Keywords:** direct reduced iron, electric arc furnace, low-CO_2_ steelmaking, submerged arc furnace, hydrogen-based steelmaking, DRI dissolution

## Abstract

Since the European Union defined ambitious CO_2_ emission targets, low-carbon-emission alternatives to the widespread integrated blast furnace (BF)—basic oxygen furnace (BOF) steelmaking strategy—are demanded. Direct reduction (DR) with natural gas as the reducing agent, already an industrially applied technology, is such an alternative. Consequently, the melting behavior of its intermediate product, i.e., direct reduced iron (DRI), in either an electric arc furnace (EAF) or a submerged arc furnace (SAF), is of great interest. Based on the conditions in these aggregates, a test series to experimentally simulate the first few seconds after charging DRI was defined. DRI samples with different carbon contents and hot briquetted iron (HBI) were immersed in high- and low-carbon melts as well as high- and low-iron oxide slags. The reacted samples were quenched in liquid nitrogen. The specimens were qualitatively evaluated by investigating their surfaces and cross sections. The dissolution of carbon-free DRI progressed relatively slowly and was driven by heat transfer. However, carbon, present either in the DRI sample or in the melt, not only accelerated the dissolution process, but also reacted with residual iron oxide in the pellet or the slag.

## 1. Introduction

In 2021, more than 1.8 billion tons of crude steel were produced worldwide [1]. The integrated route Blast Furnace (BF)—Basic Oxygen Furnace (BOF)—is still the critical steelmaking strategy, with a share of more than 70% of the global steel production. The second most important steelmaking route is the scrap-based electric arc furnace (EAF) process. However, as the availability of high-quality scrap indicates [2,3], it will not be possible to meet the demand for steel without ore-based raw materials. Due to the limited potential for reducing CO_2_ emissions of the blast furnace, new iron ore reduction technologies are required [4,5,6].

A suitable technology is the hydrogen-based direct reduction process. Direct reduction (DR) refers to a solid–gas reduction reaction [7], for example, in a shaft furnace [7,8,9,10] or a fluidized bed reactor [7,11]. The ore, in shafts, either pellet or lump ore [12,13], is converted into the so-called sponge iron or direct reduced iron (DRI) as an intermediate product, which is typically melted in an EAF for further steelmaking [14,15,16,17,18,19]. In 2020, about 104 million tons of DRI were produced, primarily based on natural gas (NG). Although this accounts only for a small share of the global crude steel production, it is a widely used steelmaking strategy, particularly but not exclusively, in NG-rich countries [16,17,20,21,22,23]. Besides the EAF, processing sponge iron into pig iron using a submerged arc furnace (SAF) might be a second option [24,25]. These aggregates are typically used to produce ferroalloys [26,27,28,29,30] and process DRI made from ilmenite- or titanomagnetite-based ores into hot metal [31,32,33]. Since it is expected that the described processing strategies will become more important, the behavior of sponge iron when immersed in liquids is of great interest for the optimization of the melting process.

The DRI properties and their influence on the EAF operation have been investigated extensively [14,15,16,34,35,36,37,38]. Some key findings are summarized below.

Cárdenas et al. [14] applied mass and energy balances to investigate various DRI scrap ratios and their influence on different process parameters. As sponge iron contained a higher proportion of oxides, the energy demand, slag quantity, and lime consumption increased. Further, a higher carbon content and a high metallization degree lowered the electrical energy consumption.

Kirschen et al. [15] described a calculation model to analyze the influence of various DRI amounts on the EAF process. One of the key findings was the increasing energy consumption with a rising DRI fraction, resulting from the larger quantity of slag and endothermic reduction reactions with unreduced iron oxide. If the the carbon content in the DRI was carefully balanced, the oxygen addition remained relatively constant, but the yield decreased with more sponge iron in the charge. Furthermore, Kirschen et al. [39] compared process data from 16 industrial EAF with varying scrap and DRI mixtures in a more recent study. Based on the results, a lower basicity is suggested to reduce the slag amount. Further, the MgO saturation must be considered when decreasing the basicity to avoid an increased wear of the refractory material. Compared to scrap charges, higher fluctuations in FeO content were measured for the slags. Possible explanations could be low metalized DRI fines and a decreased efficiency of the carbon injectors. As part of this study, the authors also optimized a previously published EAF model [40,41,42,43,44] with respect to the application of DRI.

Lule et al. [16] presented results from the ArcelorMittal Lázaro Cardenas melt shop, focusing on the behavior of nitrogen. High-carbon DRI was beneficial for making nitrogen-critical steel grades due to the extensive formation of CO bubbles.

Further, there are publications about industrial practices with high-DRI EAF charges [36,37,38]. The following general conclusions can be drawn from these publications: a higher DRI fraction resulted in a rising energy consumption, especially when the acidic gangue content increased; due to the enhanced slag volume, the iron yield decreased as more Fe was lost in the slag; regarding tramp elements such as P or Cu, an increased DRI ratio was beneficial; furthermore, the metallization of the sponge iron should be as high as possible. 

While most of the studies described above focused on industrial conditions, some publications analyzed the DRI melting and dissolution mechanisms at laboratory scale. Sharifi and Barati [45], as well as Li and Barati [46], investigated the reactions between DRI and steelmaking slags. The authors dropped DRI pellets into liquid slag pools and analyzed, e.g., the pressure increase in their furnace resulting from DRI–slag reactions. One significant result was that the decarburization involved two steps: reducing FeO in the DRI and progressing with FeO in the slag. Sadrnezhaad and Elliot [47] conducted similar experiments. Beside the gas volume, also the temperature evolution in the pellet was measured. Based on the results, the authors described the formation of a solid slag shell on a cold sample. In a further study, this idea was used by Martínez et al. [48] for the development of a melting kinetic model. The influence of carbon in the liquid on the melting behavior of solid metals was extensively investigated, e.g., by Szekely et al. [49] and Penz et al. [50,51,52]. Penz and Schenk summarized the current knowledge on this topic in a review paper [53]. In addition to determining parameters such as the heat transfer coefficient, an important phenomenological finding is the diffusion-based melting process, i.e., the diffusion of carbon from the liquid hot metal into the solid metal. This decreases the latter’s liquidus temperature, a crucial step in the interaction between scrap and hot metal in the basic oxygen furnace (BOF).

This work aimed to analyze the interaction between a DRI pellet and molten metal directly after charging. DRI samples were dipped into liquid steel, hot metal, and typical EAF and SAF slags for a specific time period. While the previous studies described above focused on industrial, carbon-containing samples, in our case, DRI with 0%C was also considered. Subsequently, the immersed specimens were visually and metallographically examined and qualitatively compared concerning the interaction behavior between the sponge iron sample and the melt. The detailed comparison of the different model cases is a novelty for studying influencing parameters such as the carbon content of the melt as well as of the DRI or the difference between slag and steel as the liquid medium.

## 2. Materials and Methods

### 2.1. Experimental Equipment

**Figure 1:**
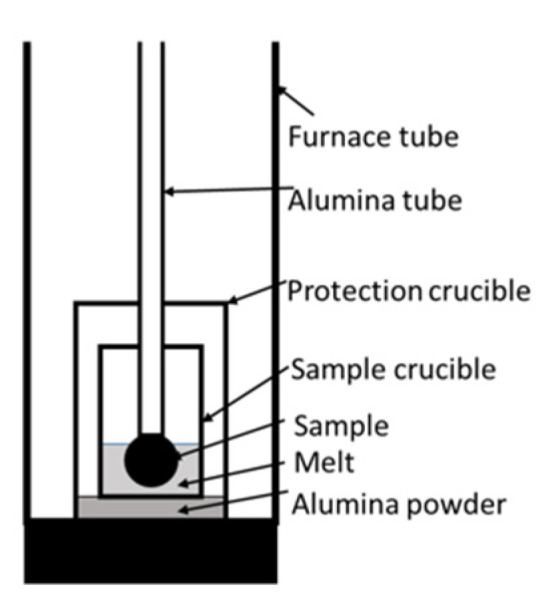
Test setup of the high-temperature tube furnace.

The experiments were carried out using a GERO^®^ HTR-V100-250/17 high-temperature tube furnace (Carbolite Gero, Neuhausen, Germany). Figure 1 shows the test setup. The tube was flushed with 350 Nl/h N_2_. An alumina protection crucible was placed in the furnace tube to avoid damage resulting from splashes. The sample crucible of MgO or alumina inside the protection crucible stood on alumina powder. A Mo wire and a screw fixed a single DRI pellet on an alumina tube. Table 1 describes detailed dimensions.

A manually operated pneumatic cylinder controlled the dipping process during the test. Every trial was filmed to evaluate the exact immersion time. After discharging, the sample was quenched via liquid nitrogen to avoid extensive reoxidation. Per each melt, three samples were immersed; after the third test, the melt temperature was measured with Heraeus Type S thermocouples (Heraeus, Hanau, Germany).

Before the metallographic preparation, we took photographs of each sample using a Sony Alpha 6000 DSLM camera (Sony Group Corporation, Tokyo, Japan). Afterward, the specimens were cold-embedded, halved, ground, and polished. The microsections were investigated using a Keyence VHX 7000 digital microscope (Keyence Corporation, Osaka, Japan). 

### 2.2. Materials

Typical BF-grade [13] iron ore pellets were used; their composition is shown in Table 2. Samples of appr. 500 g ore were prepared using a vertical reduction furnace (VRF), precisely described in [54]. After preheating under nitrogen purging with 20 Nl/min, the reduction was performed at 900 °C with 25 Nl/min pure H_2_. The furnace has a scale that allows monitoring the weight loss during reduction. The process was stopped after reaching a metallization degree of approximately 90%. The carbon-free DRI sample is called “0%C” in the following chapters.

To carburize some carbon-free DRI with Methane, approx. 130 g was recharged into the VRF and treated with 4 Nl/min CH_4_. Table 3 lists the carburizing temperatures and times, the contents of C and metallic, divalent, and trivalent iron (Fe_met_, Fe^2+^, Fe^3+^), and the metallization degree (MD). The contents of the Fe species (Fe_tot_, Fe_met_, Fe^2+^) were analyzed using titration methods, and the total carbon content was analyzed by LECO (LECO Corporation, St. Joseph, MI, USA), without focusing on its bonding state. An industry partner delivered the Hot Briquetted Iron (HBI) sample, whose chemical composition is shown in Table 3.

Ultra-low carbon (ULC) steel was used to simulate low-carbon crude steel conditions in the EAF; the samples for the melting tests were cut from a continuous casting slab provided by an industrial partner. Table 4 lists its chemical composition, analyzed by optical emission spectroscopy (OES).

For the hot metal (HM) tests, desulphurized HM chips with a diameter of 34 mm and a width of 9.4 mm were used. Table 5 summarizes the HM composition analyzed with OES.

The slags were synthetically prepared from pure oxides and premelted in an “Indutherm MU700” induction furnace (Indutherm Erwärmungsanlagen, Walzbachtal, Germany). The liquids were cast onto steel plates for rapid cooling before being used for the immersion tests. Table 6 and Table 7 report the slag compositions. The slags were dissolved in Li tetraborate and analyzed with inductively coupled plasma optical emission spectroscopy (ICP-OES).

### 2.3. Immersion Test Program

As mentioned above, two DRI process routes are possible in the future, which means the DRI production will be combined with either EAF or SAF. With this in mind, a test program was established for the following aspects of DRI melting:

- Influence of C content in DRI (carbon-free, hydrogen-based DRI was compared to carburized DRI, which approximates natural gas-based DRI)
- Composition of the liquid metal (low and high C content)
- Composition of the slag (EAF and SAF slag)
- Density of the DRI samples (DRI vs. HBI)

Table 8 sums up the executed tests with their parameters. The nomination of the sample number contains the DRI type (0%C, C750, C800, and HBI) as well as the specific melt (ULC, HM, EAF-[slag], SAF-[slag]); T_melt_ is the measured temperature of the melt after the tests. In the experiments with EAF slag, temperature measurement was impossible since an MgO crucible was used, whose inner diameter was too small to insert the thermocouple.

The samples C750-ULC-1, C800-ULC-1 and C800-ULC-2 were used as pre-tests and cooled with gaseous nitrogen. They were not further examined. The immersion time was usually 3–4 s, except for the sample 0%C-ULC-10s-3, for which it was set to 10 s to analyze the melting progression of carbon-free DRI. The accuracy was about 1 s because the pneumatic cylinder was manually controlled. The EAF slag was liquefied in an MgO crucible; all the other tests were performed in an Al_2_O_3_ crucible, as shown in Table 1.

## 3. Results

During the experiments, a few aspects were noticeable, as reported below.

In some tests with the carburized samples, sparks and splashes could be observed. This could be due to the chemical reaction between C and FeO, which generates CO gas. This phenomenon was most pronounced when DRI or HBI was immersed in hot metal. 

In all tests with hot metal (0%C-HM-1, 0%C-HM-2, and HBI-HM-1), the samples were melted entirely after a 3–4 s dipping time. As a consequence, no more investigations were possible. A further reduction of the immersion time was not manageable, since the actuator for controlling the pneumatic cylinder movement was at its limit. Another noteworthy point is the remarkable temperature difference between the furnace and the melt during the hot metal tests. The endothermic reduction reactions of FeO with C during the immersion of the samples are the most likely explanation for this phenomenon. 

After the tests, the samples were evaluated visually. The following was noticeable about the images.

**Figure 2:**
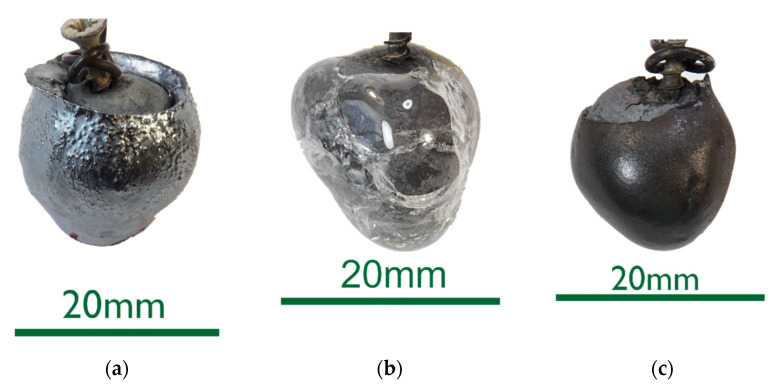
Macroscopic photographs of selected 0%C samples: (**a**) 0%C-ULC-1; (**b**) 0%C-SAF-1; (**c**) 0%C-EAF-2.

**Figure 3:**
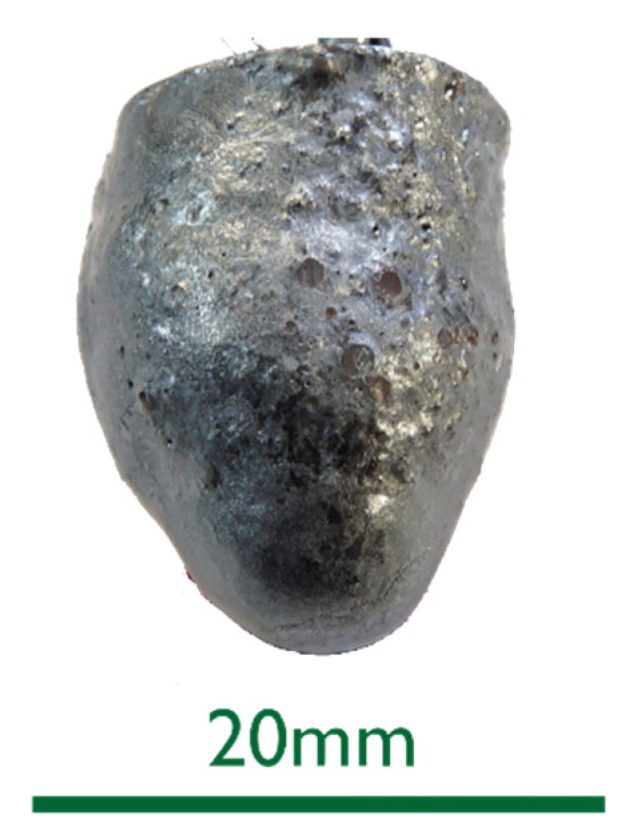
Macroscopic photograph of the sample 0%C-ULC-10s-3.

**Figure 4:**
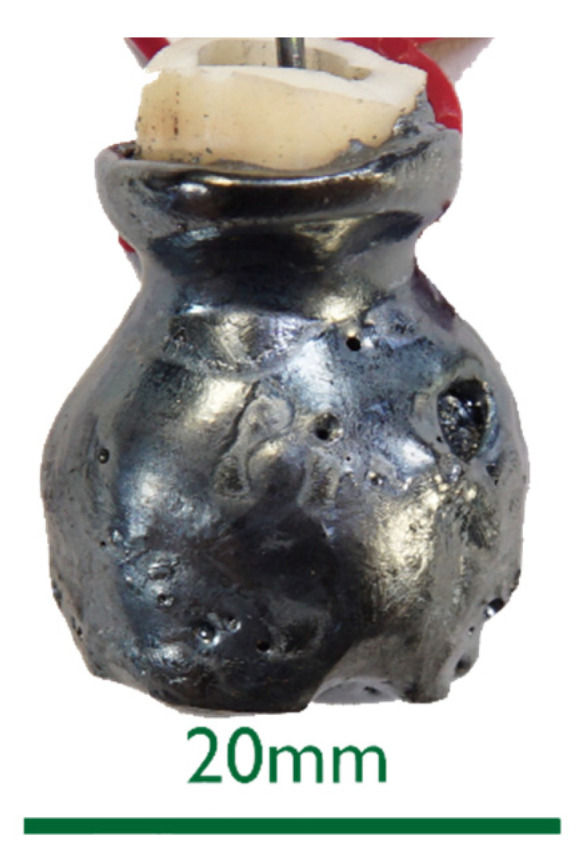
Macroscopic photograph of the sample C750-ULC-3.

**Figure 5:**
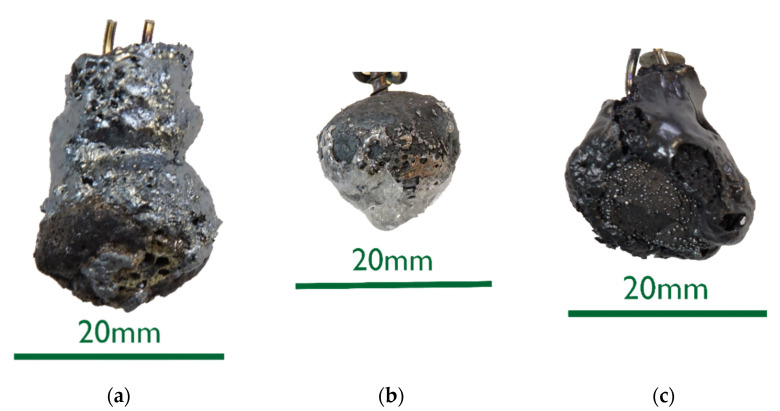
Macroscopic photographs of selected C800 samples: (**a**) C800-ULC-4; (**b**) C800-SAF-1; (**c**) C800-EAF-1.

**Figure 6:**
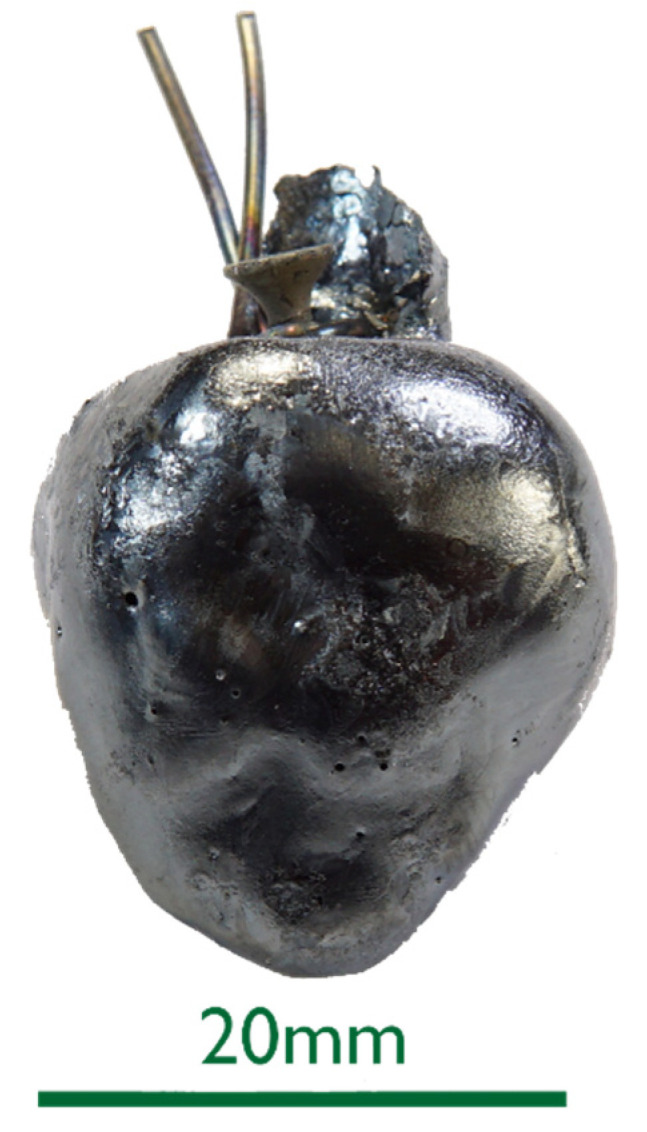
Macroscopic photograph of the sample HBI-ULC-2.

- The 0%C samples in Figure 2 showed smooth surfaces for steel and slags. This indicates that no chemical reactions took place between DRI and the liquids. After an increased immersion time, see sample 0%C-ULC-10s-3 in Figure 3, the optical impression of the surface was less shiny and appeared matte.
- As the DRI carbon content increased, a reduction of the remaining iron oxide seemed to happen. Consequently, the surface became more fissured with increasing carbon levels (compare Figure 2a with Figure 4 and Figure 5a). Frozen gas bubbles on the adhering melt residues indicated gas formation. When a highly carburized DRI-pellet was immersed in a slag containing iron oxide, a reaction of C from the sample with FeO in the slag might have occurred (see sample C800-EAF-1 in Figure 5c).
- When immersed in the SAF slag, no reactions were expected, as the temperature was much lower. The poorer slag adhesion at C800-SAF, shown in Figure 5b, indicates less wetting than for the carbon-free sample 0%C-SAF, visible in Figure 2b. This observation is consistent with slag wetting on carbon-containing refractory material [55]. Comparing the shell shapes of 0%C-ULC-1 and C750-ULC-2 in Figure 2a and Figure 4, respectively, the former was more or less droplet-shaped, while the latter had a plain surface on the bottom. This flat lower area is also visible in C800-ULC-4 and C800-EAF, as implied in Figure 5a,c. One explanation could be that the melt on the surface of the carburized pellets had a lower viscosity than the almost carbon-free ULC melt [56].
- Despite the significant carbon content of 2 wt.%, HBI showed a similar, smooth, glossy surface as 0%C-DRI (see Figure 6).

Replicated tests are not illustrated, considering the results were qualitatively reproducible. As described above, the samples dipped in hot metal were completely melted during immersion. Thus, no further analysis was possible. This rapid dissolution indicates a different melting mechanism, as the theoretical melting point of pure iron is approx. 1538 °C. The liquidus temperature was calculated using Factsage 8.0 and the FactPS database and was much higher than the HM temperature, as shown in Table 8. This effect seems analogous to diffusive scrap melting, which was extensively investigated by Penz et al. [50,51,53]. Carbon diffused from liquid hot metal to the solid iron phase at the liquid–solid interface, with heat being transported in the same direction. The high carbon content in the solid lowered its liquidus temperature and caused its further melting.

**Figure 7:**
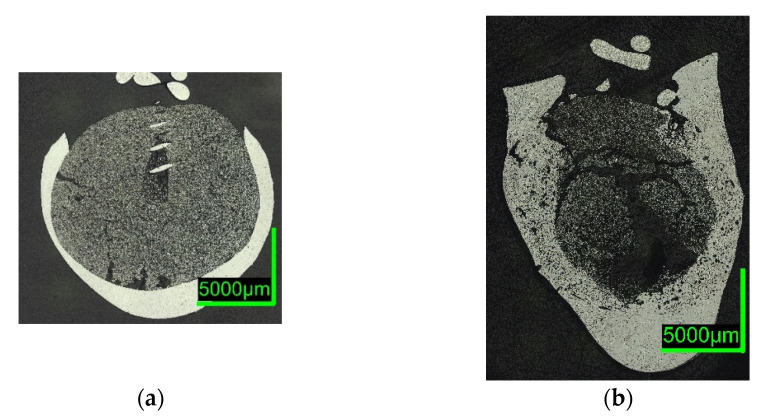
Digital microscope images of (**a**) 0%C-ULC-1; (**b**) 0%C-ULC-10s-3, dipped into liquid ULC steel.

**Figure 8:**
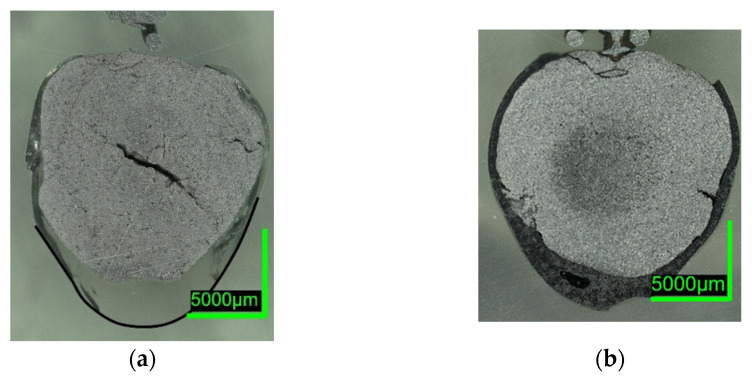
Digital microscope images of (**a**) 0%C-SAF-2; (**b**) 0%C-EAF-2, dipped into EAF/SAF slag.

**Figure 9:**
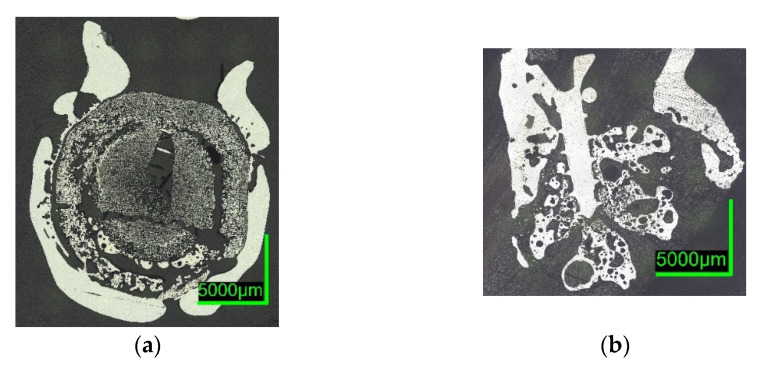
Digital microscope images of (**a**) C750-ULC-2; (**b**) C800-ULC-4, both dipped into liquid ULC steel.

Digital microscope images are shown in Figure 7 and Figure 8 below. The sections of 0%C-ULC-1 and 0%C-ULC-10s-3 with 4 and 10 s immersion time demonstrate that the pellet melted as heat transport from the surface to the core progressed (compare Figure 7a,b). Comparing the 0%C-ULC samples with C750-ULC-2 and C800-ULC-4 in Figure 9, carburized DRI appeared to liquefy much faster, since the higher carbon content lowered the liquidus temperature [53].

**Figure 10:**
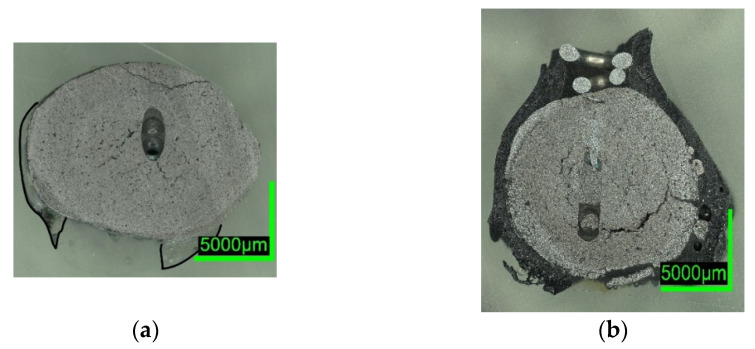
Digital microscope images of (**a**) C800-SAF-1; (**b**) C800-EAF-1, dipped into slag.

Comparing the samples immersed in slag, shown in Figure 8 and Figure 10, the results are consistent with the photographs in Figure 2b,c and Figure 5b,c. A slag layer coated the DRI surfaces; the absence of bubbles indicated that no reactions occurred when carbon-free DRI was immersed. On the other hand, carburized DRI formed a less adherent slag layer with the iron oxide-free SAF slag and was highly reactive with the iron oxide-containing EAF slag. The former phenomenon could be explained by the poor adhesion of the glassy slag layer. The latter was due to the rough surface of C800-EAF, visible in both the photograph and the digital microscope image in Figure 5 and Figure 10. Table 6 and Table 7 reports the slag composition before and after the experiments. 

**Figure 11:**
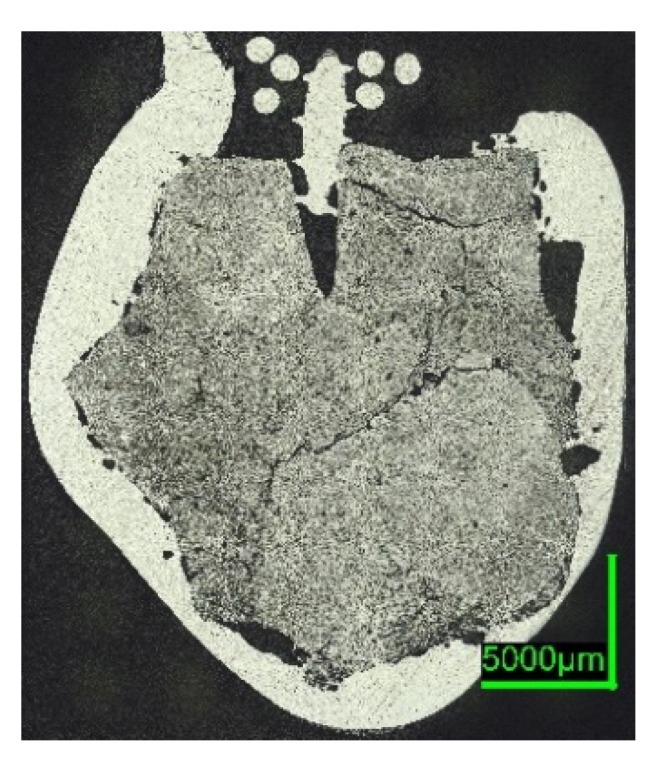
Digital microscope images of the HBI-ULC-2-1 sample, dipped into ULC.

HBI shows a behavior similar to zero-carbon DRI, suggesting that the higher density of briquetted material compared to that of unbriquetted material had a greater influence on the dissolution behavior than the carbon content in HBI. This typically ranges between 0.5 and 1.6% [57,58] or even 2% as in the present case (compare Figure 2 and Figure 7 with Figure 6 and Figure 11).

### Heat Transfer Conditions

For comparing the slag and the ULC immersion tests, differences in the heat transfer conditions should be considered. While the slag temperature in the C800-SAF test was 1479 °C, the liquidus temperature of iron with 3.79% carbon was approx. 1213 °C, calculated by FactSage^TM^ 8.0 using FSStel Database. This resulted in a significant superheating of 266 °C. As in Figure 8 no melting of the pellet can be noticed, the heat transfer could be limited when DRI became in contact with the non-reactive slag. In the scope of a similar study, Sadrnezhaad and Eliot [47] described a solid adherent slag layer. Using inert nickel spheres, the authors measured a maximum layer thickness between approximately 1.5 and 2.5 mm. A similar effect was described by Pineda-Martìnez et al. [48] by modeling the heat transfer and comparing it to literature data.

The Prandtl (Pr) numbers were calculated to characterize the slag and the ULC melt conditions. Pr, shown in Equation (1), describes the ratio between the velocity boundary layer and the temperature boundary layer, calculated by the kinematic viscosity ν/(m^2^/s) and the thermal diffusivity a/(m^2^/s). Transforming both components, Pr can be written as a function of the kinematic viscosity η/(Pas), the heat capacity of the fluid cp/(J/Kmol), and λ_Fl_ [59,60].

(1) $$\mathrm{Pr}=\frac{\nu }{a}=\frac{\eta \cdot \mathrm{cp}}{\lambda_{\mathrm{Fl}}}$$

Table 9 lists the calculated values and the used parameters for the previously mentioned Prandtl number for both cases, slag–DRI and liquid iron (ULC)–DRI. It shall be noted that some literature data vary broadly. Nevertheless, the results indicate the order of magnitude. The lower thermal conductivity and the higher viscosity of liquid slag, compared to liquid iron, result in a Prandtl number which is increased by a factor of 1000 (176 vs. 0.111). Consequently, when in contact with slag, the temperature boundary layer, an indicator for thermal diffusion, is relatively thin compared to the velocity boundary layer, representing impulsive transport. This difference may be a reason for the unmelted carburized DRI, despite the relatively high superheating.

## 4. Discussion

ULC experiments: The results of dipping 0%C samples and HBI into a low carbon melt indicated that heat transfer was the driving force in this case. The 0%C-ULC-3 specimen in Figure 7b confirmed this, since it showed the growth of a liquefied shell. The higher the carbon content, the thicker the shell at similar dipping times. The lower liquidus temperature explained the faster melting in iron–carbon mixtures. In principle, the observations agree with the calculated results of González et al. [62], i.e., an increasing sample diameter immediately after immersion, followed by the melting of this surface layer. However, the melting time strongly depend on parameters such as DRI porosity, initial diameter, or gangue content [62]. Therefore, it was difficult to make quantitative comparisons. Additionally, the high-carbon samples in Figure 4 and Figure 5 had a rough surface with many bubbles due to the reduction of some residual iron oxide with carbon. These reactions, along with the resulting blisters, may benefit the EAF process. Various authors [16,34,38] reported benefits in terms of lower electrical energy consumption, less nitrogen content, or better slag foaming.

Hot Metal experiments: The DRI and HBI samples were fully melted after 3–4 s of immersion in the hot metal. At first sight, this was not expected, as the liquidus temperature of pure iron (approx. 1538 °C, according to FactSage^TM^ 8.0 and the FactPS database) was higher than the hot metal temperature. Further, this differs from the results for ULC steel. Penz et al. [50,51,52,53] investigated the dissolution of scrap in hot metal, which is a similar process, and described the following melting steps:

- Initial formation of a solid hot metal layer, which liquefied again after the heat transport provided enough overheat [53]
- Diffusive melting meant a mass transfer from carbon-rich hot metal to low-carbon-containing solid steel [53]
- Heat transfer was the primary mechanism if the carbon content of both phases was balanced or the scrap temperature exceeded its liquidus temperature [53]

Slag experiments: No reactions occurred, since no iron oxide was available in contact with SAF slag. The high C sample C800 was conspicuous in two respects: first, the slag layer adhered only loosely, indicating less wetting than on the 0%C-sample; second, the entire pellet appeared unmelted, which was unexpected, as the melting temperature of the carburized iron was 266 °C below the slag temperature. In comparison, the ULC melt of the test C750-ULC-3 was approx. 143.5 °C (T_ULC_ = 1544 °C; T_liq_ = 1400.5 °C, acc. to FactSage^TM^ 8.0, FactPS, FSStel databases) overheated and was liquefied in the near-surface region. Therefore, the Prandtl number was calculated for both ULC and slag cases. In the latter case, it was approx. 1000 times higher, indicating different heat transition conditions. This observation was in qualitative agreement with the numerically calculated results of González et al. [62] and Pineda-Martínez et al. [48], who reported significantly longer melting times in contact with slag.

In contrast, the high-C sample appeared to be very reactive in contact with the EAF slag, which contained high levels of iron oxide. The core of the DRI pellet, see the microscope image in Figure 10b, was almost unmelted, consistent with C800-SAF and indicative of lower heat transfer when in contact with a slag. Nevertheless, bubble formation due to the reaction between carbon and iron oxide could have led to higher turbulence in the surface boundary layer and thus an increased heat transition.

## 5. Conclusions

A vertical furnace was used to investigate the behavior of sponge iron in contact with liquids. High, medium, and carbon-free-DRI, as well as HBI, were immersed into melts of ULC, hot metal, and slags. Based on metallographic examinations, processes occurring in different cases were characterized. Carbon, either in the sponge iron or in the melt, increased the melting rate due to its effect on the liquidus temperature of iron. Further, reduction reactions of iron oxides, either in the DRI pellet or in the high-FeO slag, could be observed, as indicated by the occurrence of gas bubbles on the surfaces of these samples. Even the high-carbon DRI sample remained unmelted when in contact with iron oxide-free slag. This indicated different heat transfer conditions between liquid slag and steel, which was confirmed by calculating the Prandtl numbers. While HBI also showed a rapid dissolution in hot metal, the behavior in a low-carbon melt was the same as for 0%C-DRI, that of sponge iron without carbon. Therefore, particle density was also a crucial parameter in the melting behavior.

## Figures and Tables

**Table 1:** Characteristics of the test setup components.

Component	Dimensions/mm	Material	Comment
Protection Crucible *	Ø117.6 × Ø108.6 × 180	Al_2_O_3_	
MgO Sample Crucible *	Ø48.5 × Ø36.0 × 105	MgO	for EAF slag test
Alumina Sample Crucible *	Ø49.5 × Ø42.6 × 68.7	Al_2_O_3_	for ULC, HM, and SAF slag tests
Wire	Ø1	Mo	
Screw	“Spax” 2.5 × 12	steel	
Furnace Chamber	Ø180	Al_2_O_3_	

* one exemplary sample was measured.

**Table 2:** Composition of the unreduced ore pellets in wt.%.

Fetot	Fe_2_O_3_	FeO	CaO	SiO_2_	Al_2_O_3_	MgO
64.9	92.5	0.37	0.48	4.55	0.84	0.45

**Table 3:** Carburizing conditions and composition of the DRI and HBI samples/species in wt.%.

Sample	T_Carb_/°C	t_Carb_/min	Fe_met_	Fe^2+^	Fe^3+^	C_tot_	MD/%
C750	750	20	81.35	5.01	0.01	1.79	94.2
C800	800	25	82.46	2.82	0.00	3.71	96.7
HBI	-	-	84.6	4.1	2.3	2.00	93.0

**Table 4:** ULC composition/wt.%.

C	Si	Mn	Al	Ti	S	P	Ni	Cu	Cr
0.007	<0.001	0.146	0.028	0.079	0.015	0.009	0.011	0.005	0.027

**Table 5:** HM composition/wt.%.

C	Si	Mn	S	P
4.6	0.4	0.6	0.004	0.07

**Table 6:** SAF-like slag composition in wt.%; all values were measured as elements and converted to oxides.

	Before Test	After Test
CaO	40.1	39.4
SiO_2_	39.8	39.1
Al_2_O_3_	11.3	12.9
MgO	8.02	7.80
Fe	0.62	0.62
B2 = CaO/SiO_2_	1.01	1.01

**Table 7:** EAF-like slag composition in wt.%; all values were measured as elements and converted to oxides.

	Before Test	After Test
CaO	27.2	18.8
SiO_2_	21.2	13.4
Al_2_O_3_	8.20	9.73
MgO	9.88	31.2
Fe	26.1	20.9
B2 = CaO/SiO_2_	1.28	1.40

**Table 8:** List of the samples.

Sample Number	Liq. Medium	t_Immersion_/s	T_furnace_/°C	T_melt_/°C
C750-ULC-1 *	ULC	4	1625	-
C750-ULC-2		3		1544
C750-ULC-3		4		1555
C800-ULC-1 *		4		-
C800-ULC-2 *		3		-
C800-ULC-3		3		1555
C800-ULC-4		4		1555
0%C-ULC-1		4		1544
0%C-ULC-2		3		1544
0%C-ULC-3-10s		10		1558
HBI-ULC-1		3		1558
HBI-ULC-2		3		1558
0%C-HM-1	HM	4	1500	1390
0%C-HM-2		3		
HBI-HM-1		3		
0%C-SAF-1	SAF slag	3	1550	1479
0%C-SAF-2		3		
C800-SAF-1		3		
0%C-EAF-1 ^x^	EAF slag	3	1600	-
0%C-EAF-2 ^x^		3		
C800-EAF-3 ^x^		3		

* samples used as pre-tests, which were cooled via nitrogen flushing; ^x^ no temperature measurement was possible, as the MgO crucible was too small to insert the thermocouple.

**Table 9:** Calculated dimensionless Prandtl numbers, incl. literature data for the parameters.

Parameter	Value	Comment	Source
cp_Fe,liq_	820 J/kgK		[48]
λ_Fe,liq_	37.65 W/mK		[48]
η_Fe,liq_	5.08 mPas	at 1550 °C	[56]
cp_slag_	696–1171 J/kgK	696 used for calculation	[48,61]
λ_slag_	1.1715–1.3589 W/mK	1.2625 used for calculation	[48]
η_slag_	0.320 Pas	calculated with Factsage^TM^ for 1479 °C	
Pr_Fe-DRI_	0.111		
Pr_Slag-DRI_	176		

## Data Availability

The data presented in this study are available within the article.
